# Assessing the application of big data technology in platform business model: A hierarchical framework

**DOI:** 10.1371/journal.pone.0238152

**Published:** 2020-09-24

**Authors:** Xiaomin Du, Yang Gao, Linlin Chang, Xiangxiang Lang, Xingqun Xue, Datian Bi

**Affiliations:** 1 Department of Economic Management, Yingkou Institute of Technology, Yingkou, China; 2 School of Business, Dalian University of Technology, Panjin, China; 3 School of Management, Jilin University, Changchun, China; University of Pisa, ITALY

## Abstract

This research aims to create a hierarchical framework for the development of a platform business model based on big data. However, this hierarchical framework must consider unnecessary attributes and the interrelationships between the aspects and the criteria. Hence, fuzzy set theory is used for screening out the unnecessary attributes, a decision-making and trial evaluation laboratory (DEMATEL) is proposed to manage the complex interrelationships among the aspects and attributes, and interpretive structural modeling (ISM) is used to divide the hierarchy and finally construct a hierarchical framework. The results reveal that (1) value proposition and community building in value production are fundamental links; (2) information technology and information management in value production are technical supports; (3) customer development in value marketing is the power source; and (4) value acquisition is the last link, which is established on the basis of and influenced by value marketing and value network. This hierarchical framework aims to guide the platform toward the application of big data. This study also proposes engagement of stakeholders for promoting value creation and establishing a sound business model from multiple levels and links.

## 1. Introduction

With the development, popularization and application of information technology, the use of big data has penetrated the daily lives of ordinary people and created unprecedented opportunities for enterprises to use their data assets to conduct market activities. Google, Amazon, Facebook and other companies have undertaken great efforts in industrial operations by collecting and utilizing big data [1]. Determining how to use big data to achieve their own leapfrog development over competitors has gradually become the main task of platform enterprises.

Currently, big data is such a popular topic that many scholars have devoted themselves to studying it. Research on the application of big data in platform enterprises has mostly focused on the impact on a certain link in the value chain of platform enterprises, such as the application of big data in inventory management [2], transportation logistics management [3] and supply chain management [4]. Studies on the combination of big data and customer management are more common. Platform enterprises can use product search information to analyze consumer preferences to conduct personalized precision marketing [5]; consumers can visit different online stores to compare prices for the same product and thus influence product pricing [6]. Enterprises use big data to analyze the impact of customer online reviews on product experience to predict trends in product design innovation [7]. Although the above studies have different emphases, they ignore the mutual coordination and limitations among different links of the value chain, which is not conducive to the overall grasp of the operation of platform enterprises and is more detrimental to the overall design of the platform business model. In addition, in terms of the application of big data in platform enterprises, most studies have adopted the method of case analysis and focused on well-known platform enterprises, such as Amazon [8], JD [9] and Taobao [10], lacking a universal integration framework. E-commerce platform is a virtual network used to carrying out transactions over the Internet and a management environment for ensuring a smooth business operation. It is an important platform for the orderly coordination and integration of information flow, goods flow and capital flow. Enterprises and merchants can make full use of the network infrastructure, payment platform, security platform, management platform and other shared resources provided by E-commerce platform to carry out their own business activities more effectively and at a lower cost [11]. Therefore, it is of great significance to explore the influence of big data on the E-commerce platform business model, explore the mutual cooperation and limitations between different modules of the platform business model, and finally reveal the hierarchical path of platform enterprises under big data to compensate for the shortage of such research in the extant literature.

Based on this, the research mainly solves the following problems. First, it need to clarify the difference between the platform business model and the traditional business model. By means of literature collection, this paper follows the description of the traditional business model by Teece [12] and explores the differences between various value sectors of the platform business model and traditional enterprises from the value perspective. Then, the existing research on the business model of platform enterprises is systematically sorted, and the value plates are sorted into five categories: value proposition, value production, value marketing, value acquisition and value network. Second, this paper must address the upgrades and changes brought about by big data to the platform business model, in order to build a new business model system of platform based on big data. According to the value chain theory, this paper systematically sorts the data collected by platform enterprises into different links of the value chain and analyzes their specific applications to further summarize the impact of big data on the platform business model. Then, the new criteria system of the platform business model based on big data have been obtained. Finally, this paper must explore the development path of the platform business model under big data and build a hierarchical framework. In this process, the dependence among criteria should be identified. Then, this research considers the mutual restrictions and influences among criteria and finally makes multi-attribute decisions under multiple criteria. This paper uses fuzzy set theory and decision-making and trial evaluation laboratory (DEMATEL) to evaluate the cause-and-effect relationships among various criteria and probes into the degree of comprehensive influence among various criteria. At last, this study uses an interpretive structure model (ISM) to divide the hierarchy and finally construct a hierarchical framework of the platform business model under big data.

The following conclusions are found in this paper. First, the influence of big data on the platform business model is mainly reflected in five aspects: value proposition, value production, value marketing, value acquisition and value network. Second, we find that these five aspects are in different positions and have complex relationships with each other: (1) value proposition and community building in value production are at the first level, and both have the most important impact on the later levels. (2) Information technology and information management in value production are at the second level, which becomes the subsequent point for platform enterprises to attract customers. (3) The criteria of value marketing and value network are almost at the third and fourth levels; they influence and restrict each other and are the last links before value acquisition. (4) Value acquisition at the last level requires the joint promotion of the first four levels. This paper not only makes up for the shortage of research on the use of big data to improve the competitiveness of platform enterprises but also constructs a hierarchical framework for the platform business model based on big data for the first time, which provides guidance for platform enterprises to use big data to establish a sound business model and obtain competitive advantage.

## 2. Literature

### 2.1 Big data

Over the past few decades, technological development has dramatically expanded the amount of data available to an organization, enhancing the importance of data and information to enterprises competitiveness [13, 14]. Therefore, scholars have paid more attention on how enterprises can use big data to create value [15]. Wamba et al. [16] define big data as an integral approach to manage and analyze five Vs (i.e. volume, variety, velocity, veracity and value) so as to create feasible insights for sustainable value delivery and establishing competitive advantages. Big data can change competition among enterprises by “transforming processes, altering corporate ecosystems, and facilitating innovation” [17]. Besides, it can unlock organization business value by unleashing new organizational capabilities and new value, which is useful to tackle their key business challenges [18]. At a moment when the survival of E-commerce platform is threatened by the highly volatile economic conditions, Big Data can remodel the business model and provide a much-needed competitive edge which can improve profitability and the chances of survival [19, 20].

### 2.2 Traditional business model

There are many studies of traditional business models among scholars, and scholars have given definitions of business models from various perspectives. The business model is understood as an overall description of how an enterprise creates value through interdependent activities in its business ecosystem [21]. Timmers [22] proposed that the business model includes products, the architecture of service, information flow, business participants and their roles, participants’ interests, revenue sources and marketing strategies. Osterwalder [23] initially identified the nine elements that constitute the business model. Johnson and Christensen [24] stated that the business model is composed of four interacting factors: customer value proposition, key resources, key processes and profit model. Teece [12] described the business model as "the design or architecture of the value creation, delivery, and capture mechanisms". Through the summaries of the business model by existing scholars, it can be found that the key elements of the business model mainly focus on "value proposition", "value creation", "value delivery" and "value acquisition".

### 2.3 Platform business model

Platform business model can be thought of as an open business model, with the openness of the multilateral networks including platform users, platform infrastructure, platform providers and so on [25, 26]. Multilateral networks allow platform enterprises to link various groups of participants through a highly adaptable and permeable infrastructure, which makes it possible to enable information and knowledge flow throughout the network of multilateral participants [27]. In the network era, benefits are increasingly generated through platforms, which allow various participants to engage with one another [28]. This novel form of participant-to- participant service exchange challenges the idea of one firm managing an entire activity system—an idea nested in traditional business model [29].

There are some differences between the platform business model and the traditional business model. The model expression of traditional enterprises attaches importance to the induction and refinement of the components of the business model but ignores the analysis of internal relations among the components of the business model as a whole [30]. Weiblen [31] claims that the business model of platform enterprises will be more open, collaborative, competitive and networked in the future, thus forming a more sustainable and stable platform network ecosystem. Evans [32] state that the scope and depth of the bilateral network effect and the differentiated distribution of bilateral consumers are the key factors that determine the success of platform enterprises’ operations. The description of the constituent elements and the relationship between these elements are two basic levels for understanding the platform business model [8]. This paper finally concludes the five major parts of the platform business model, namely value proposition, value production, value marketing, value acquisition and value network.

### 2.4 The influence of big data on the platform business model

Recently, big data has attracted extensive attention from academic circles [33, 34]. The emergence of big data has made a significant impact on the development of high-tech platform enterprises [35] and has promoted the transformation of enterprises from being traditional-factor driven to being innovation driven [36]. Platform enterprises can use the large amount of content created by the platform to excavate hidden opportunities, continuously develop new products, new technology and new services, and thus enhance their competitive advantage [37].

#### 2.4.1 Value proposition

McKinsey initially defined value proposition as "the benefits provided to the customer community and the price that the customer will pay" [38]; that is, value proposition is the description of the content of the value provided by the target customer [39]. Dibb [40] introduced the concept of "market segmentation." The essence of market segmentation is to aggregate convergent consumers in the market environment and define the target consumer group [41]. Against the background of big data, platform enterprises can understand customer needs according to their browsing and purchasing conditions and determine target customers based on the enterprise’s business strategy [42]. In addition, as technology advances, the digital transformation of the business model is reshaping consumer preferences [43], so enterprises must adjust their value content according to the dynamic changes in the industry to enhance their competitiveness. The platform can use the customer evaluation content to determine customer preferences and decide whether to develop its own products or its own logistics system to make adjustments to its own value content [44]. This paper divides the value proposition of platform enterprises based on big data into two secondary criteria: market segmentation and value content.

#### 2.4.2 Value production

Value production involves a company’s value structure and mechanism, which are reflected in the arrangement of enterprise resources and processes [45]. Platform enterprises use electronic processing and information technology as the bases of their information transmission tools and use electronic means to engage in business operations and sales activities [46]. In the context of big data, technical support becomes more important. Platform enterprises use web or mobile application technology to collect accurate and effective data to better analyze customer preferences [47]. Currently, the key to attracting a large number of customers involves the different experiences brought about by different interface structure designs and the functions of different platforms. Based on big data, platform enterprises use web mining technology to expand the keywords input by users effectively and quickly to improve the accuracy of commodity information retrieval. In addition, enterprises dynamically adjust the layout and classification of the entire platform interfaces according to customers’ consumption habits so that customers can find the consumer goods they want at a glance to achieve the goal of meeting customers’ personalized needs efficiently [48]. Moreover, it is more convenient to use big data to manage the information content resources of suppliers, consumers and other stakeholders [49]. Enterprises can use big data to precisely classify user data, thus facilitating precision marketing [50]. This paper divides the value production of platform enterprises based on big data into three secondary criteria: technical support, community building and information management.

#### 2.4.3 Value marketing

The emergence of big data requires platform enterprises to transform their extensive marketing models into effective precision marketing models [50]. Relying on modern information means, precision marketing comprehensively and systematically analyzes the specific needs of users through the application of massive data on the platform to accurately locate customer groups and build user labels that predict customer needs [51], ultimately forming a personalized service system to attract customers. Vendrell-Herrero argues that the digital transformation of business models is reshaping consumer preferences and behavior [43], thus also changing customer relationships [52]. Then, the information of consumer groups is managed, and based on this fact, the platform has an in-depth understanding of the consumer psychology of users, which can help the platform develop personalized marketing plans to attract customers and drive existing customers to recommend the platform to potential customers [49]. This paper divides the value marketing of platform enterprises based on big data into three secondary criteria: customer attraction, relationship management and customer development.

#### 2.4.4 Value acquisition

Value acquisition describes how an enterprise converts the value it delivers to its customers into revenue and profit [12, 53, 54]. Against the background of big data, one important income stream comes from platform enterprises using their own platforms to attract advertising investors [55]. However, platform enterprises should not ignore the consumer experience purely for the sake of advertising fees. Too much advertising will make consumers feel bored and will be counterproductive [56]. Pricing strategy will also affect the revenue source of the platform, and the platform can develop personalized pricing methods based on consumer preference prices [6]. With the widespread application of big data, mobile payment services have brought about great development opportunities to platform enterprises for value acquisition. Platform enterprises can take advantage of consumer payment habits to develop financial products and strive to provide consumers and retailers with greater value than that provided by traditional payment providers (such as banks) [57]. Through the use of big data, on the one hand, enterprises can perform qualitative and quantitative assessments of buyers’ credit according to their consumption behaviors; on the other hand, enterprises can make the same assessments of seller credit by means of tracking the sales volume and service quality of merchants. With the help of this two-way credit rating system, we can better maintain and optimize the rules and regulations of buyers and sellers in the transaction process [58]. This paper divides the value acquisition of platform enterprises based on big data into three secondary criteria: media advertising, pricing and financial products.

#### 2.4.5 Value networks

Value networks constitute a unique attribute of platform enterprises. The ultimate goal of platform enterprises is to build a value network ecosystem and gather all participants in the platform to form an economic community [59]. Currently, many platforms present the phenomenon that new services or new businesses are increasing, and the scale of platforms is expanding continuously. Platform enterprises adopt the services provided by new partners with an open attitude and create new value [60]. However, data resources are now in "rampant expansion", so when platform enterprises are building the value network, legitimacy construction is necessary for almost every important decision [11]. Enterprises should strengthen their own data security construction and establish multidimensional protection measures and trustworthy protection mechanisms to ensure the confidentiality of user resources in the process of big data platform cooperation and to prevent data leakage [61]. In addition, since platform enterprises have a full and accurate grasp of data and information, they should take the initiative to assume social responsibility and combine big data with the needs of the public to better serve society as a whole. This paper divides the value network of platform enterprises based on big data into three secondary criteria: symbiosis, legality and privacy security. A detailed explanation of each criterion is shown in Table 1.

**Table 1:** Proposed attributes.

Aspects	Criteria	Explanation
Value proposition	Market Segmentation(C1)	Determine target customers according to customers’ browsing and purchasing conditions
	Value Content(C2)	Decide whether to develop their own products or own logistics systems according to the evaluation of customers on the purchasing of goods
Value production	Information Management(C3)	Management and systematic analysis of consumer information and store information
	Community Building (C4)	Design pages according to customers’ browsing habits and select suppliers according to customers’ complaint rates
	Technical Support(C5)	Improve the platform technology according to customers’ platform experiences
Value marketing	Customer Attraction(C6)	Select the optimal advertising strategy according to the promotion data of the cooperation platform and make recommendations based on the products that customers browse, click, add to the shopping cart and purchase on the platform
	Relationship Management(C7)	Use big data to establish a membership system for customer relationship management and improve corresponding services to maintain customer relationships according to customer feedback information
	Customer Development(C8)	Existing customers recommend the business to potential customers
Value acquisition	Pricing(C9)	According to the feedback of customers on product preference and price information, product pricing can be conveyed to merchants or self-operated products
	Media Advertising(C10)	Arrange advertising space according to product popularity and charge a commission in proportion to sales volume
	Financial Products(C11)	Develop financial products according to payment methods and establish credit compensation systems according to the default rates and repayment delay times of financial products
Value network	Privacy Security(C12)	Use authentication techniques to ensure the authenticity and confidentiality of partners
	Symbiosis(C13)	Select platform cooperation products and promotion modes according to platform market segmentation data
	Legality(C14)	Establish effective information security for a large amount of user information held by the platform to prevent information leakage

## 3. Method

The objective of this paper is to provide a hierarchical framework for the application of big data in the field of E-commerce platform. DEMATEL determines the importance and cause-and-effect relationships between criteria from a micro point of view. However, it cannot display the intrinsic relationship and the division of the hierarchical structure, making it is difficult to effectively manage and control these criteria [62]. ISM is macroscopically oriented and used to decompose complex systems into subsystems. ISM can transform complex thoughts and ideas into an intuitive model of structural relationships to understand the relationship between the variables. The combination of the two methods can make the results more accurate and intuitive [63]. In addition, by using fuzzy set theory, triangular fuzzy number is used to replace the original accurate value of expert evaluation, which can improve the credibility of the analysis results and provide a more valuable reference for managers to make decisions [62]. The following sections of this paper provides corresponding formulas to help to understand the integration method.

### 3.1 Fuzzy DEMATEL

DEMATEL was first proposed by Gabus &Fontela, scholars in Battelle Laboratory. DEMATEL is a systematic analysis method by using graph theory and matrix tools to handle complex and difficult problems. Through the logical relationship and direct influence matrix in the system, we can calculate the causality and centrality of criteria as the basis for constructing the model and determine the causal relationship between criteria and the position of each criterion in the system. Triangular fuzzy number (TFN) provides an effective means of quantifying human linguistic preferences into computable form. Fuzzy-DEMATEL method retains the practical and effective advantages of traditional DEMATEL method in factor recognition, while fuzzy concepts allow the capture of artificial deviations and uncertainties that DEMATEL cannot handle in the data. Therefore, Fuzzy-DEMATEL was used in this study to explore the causal relationship between the criteria and the degree of influence. The steps are as follows.

Step 1: For the problem under study, build a system of influencing factors set to F_1_, F_2_, …, F_n_.
Step 2: Determine the influence relationship between two factors by an expert scoring method and express the relationship in matrix form. Invite experts to use the language operators "no impact (N)", "very weak influence (VL)", "weak influence (L)", "strong influence (H)", and "very strong influence (VH)". The relationship between the two factors is assessed. Convert the original expert evaluations into triangular fuzzy numbers via a semantic table $w_{ij}^{k}=(a_{1ij}^{k},a_{2ij}^{k},a_{3ij}^{k})$ to represent the extent to which k experts consider the influence of the i-th factor on the j-th factor, as shown in Table 2.
Step 3: Using the Converting the Fuzzy data into Crips Scores (CFCS) method to defuzzify the initial values of the expert scores, the nth order directly affects the matrix Z, and the direct influence matrix reflects the direct effect between the factors, including the following four steps:
(1) Normalize triangular fuzzy numbers:
  $$xa_{1ij}^{k}=(a_{1ij}^{k}-\text{min}a_{1ij}^{k})/\Delta_{\text{min}}^{\text{max}}$$ (1)
  $$xa_{2ij}^{k}=(a_{2ij}^{k}-\text{min}a_{1ij}^{k})/\Delta_{\text{min}}^{\text{max}}$$ (2)
  $$xa_{3ij}^{k}=(a_{3ij}^{k}-\text{min}a_{1ij}^{k})/\Delta_{\text{min}}^{\text{max}}$$ (3)
(2) Normalize the left value (ls) and right value (rs):
  $$x\;ls_{ij}^{k}=xa_{2ij}^{k}/(1+xa_{2ij}^{k}-xa_{1ij}^{k})$$ (4)
  $$x\;rs_{ij}^{k}=xa_{3ij}^{k}/(1+xa_{3ij}^{k}-xa_{2ij}^{k})$$ (5)
(3) Calculate the clear value after defuzzification:
  $$x_{ij}^{k}=[x\;ls_{ij}^{k}(1-x\;ls_{ij}^{k})+x\;rs_{ij}^{k}x\;rs_{ij}^{k}]/[1-x\;ls_{ij}^{k}+x\;rs_{ij}^{k}]$$ (6)
  $$z_{ij}^{k}=\text{min}a_{1ij}^{k}+x_{ij}^{k}\times \Delta_{\text{min}}^{\text{max}}$$ (7)
(4) Calculate the average clear value:
  $$z_{ij}^{k}=(z_{ij}^{1}+z_{ij}^{2}+\cdots \text{+}z_{ij}^{k})/n$$ (8)
Step 4: Normalize the direct influence matrix Z to obtain the standardized direct influence matrix G:
  $$\lambda =1/\underset{1\leq i\leq n}{\text{max}}\sum_{j=1}^{n}z_{ij},G=\lambda Z$$ (9)
Step 5: According to *T* = *G* + *G*^2^ +⋯*G*^*n*^ or *T* = *G*(*E* − *G*)^−1^, E is the identity matrix, and the comprehensive influence matrix T is obtained.
Step 6: Analyze the comprehensive matrix to reveal the internal structure of the system. The elements in matrix T are added by row as the influence degree Di, which represents the comprehensive influence value of the row factor on all other factors. The elements in matrix T are added as the affected degree Ri by column, indicating the comprehensive influence value of all other factors in that column. The formulas are as follows:
  $$Di=\sum_{j=1}^{n}t_{ij}(i=1,2,\cdots ,n)$$ (10)
  $$Ri=\sum_{i=1}^{n}t_{ij}(i=1,2,\cdots ,n)$$ (11)

**Table 2:** Semantic transformation table.

Linguistic variables	TFN
N (No influence)	(0,0,0. 2)
VL (Very low influence)	(0,0. 2,0. 4)
L (Low influence)	(0. 2,0. 4,0. 6)
H (High influence)	(0. 4,0. 6,0. 8)
VH (Very high influence)	(0. 8,1,1)

The sum of the influence degree and affected degree is called centrality, which indicates the position of the factor in the system and the size of its role. The difference between the influence degree and the affected degree is called causality, which reflects the causal relationship between the influencing factors. If the causality is greater than 0, the factor has a great effect on other factors and is called the factor of cause. If the causality is less than 0, the factor is greatly affected by other factors and is called the factor of result. The formulas are as follows:

$$m_{i}=D_{i}+R_{i}(i=1,2,\cdots ,n)$$ (12)

$$\begin{array}{l} n_{i}=D_{i}-R_{i}(i=1,2,\cdots ,n)\\ H=T_{i}-R_{i}(i=1,2,\cdots ,n) \end{array}$$ (13)

### 3.2 ISM

ISM was developed in 1973 by Professor Walter Felter as a method of analyzing problems related to complex socio-economic systems, which could make full use of people’s practical experience and knowledge to decompose the complex system into several subsystems, and finally construct the system into a multi-level hierarchical structure model. As a conceptual model, ISM can transform ambiguous ideas and views into intuitionistic structural model. It is suitable for systematic analysis with many variables, complex relationships and unclear structures. The formula used to divide the hierarchy is shown below.

The comprehensive influence matrix T above reflects only the mutual influence relationship and degree between different factors and does not consider the influence of factors on itself. Therefore, it is necessary to calculate the overall influence relationship reflecting system factors, i.e., the overall influence matrix. The calculation formula is:

$${H=T+E=h}_{ij}$$ (14)

Next, a threshold λ is introduced to eliminate redundant information and obtain the most simplified matrix. According to the trial calculation, the most suitable threshold calculation model is obtained.

λ=α+β (15)

where α and β are the mean and standard deviation of all elements in the comprehensive influence matrix T, respectively.

The threshold λ is used to remove the redundant factors, and the reachable matrix is obtained.

$${M=[m}_{i}{}_{j}{]}_{{}_{n*n}}^{},(i=1,2\ldots .n;j=1,2\ldots .n)$$ (16)

$$m_{i}{}_{j}=\{\begin{array}{l} 1,h\geq \lambda \\ 0,h\leq \lambda \end{array}(i=1,2\ldots .n;j=1,2\ldots .n)$$ (17)

1 means there is a direct effect between the two factors, and 0 means there is no direct effect between the two factors.

The reachable set *L*(*f*_*i*_), antecedent set *P*(*f*_*i*_) and common set

$$C(f_{i})=L(f_{i})\cap P(f_{i})$$ (18)

are obtained by hierarchical processing.

Finally, the ISM is determined by the reachable set and common set.

## 4. Results

Based on the literature reviews and analysis, this paper summarized 14 criteria. In order to standardize the application of platform business model and ensure the embedding of big data, it is necessary to evaluate the rationality and standardization of these criteria through the expert committee which is composed of 7 experts. We have clear requirements for experts. We define the scope of experts in university scholars and e-commerce platform staffs. For university scholars, professors who have studied the same area for at least eight years were selected. For e-commerce platform staffs, we mainly select middle and senior leaders who have good knowledge of business model and rich experience in practice.

Before evaluating the attributes proposed in this study (including aspects and criteria), the expert committee should prove these attributes can reflect the real situation of the platform enterprises. Once one expert disagrees with the proposed attributes, the committee needs to discuss this problem until all experts reaching an agreement. Therefore, several rounds of discussion would be needed to ensure the reliability of this research. Once the criteria are confirmed, the questionnaire used to evaluate the importance of the criteria was conducted. Then, we sent out questionnaires to each expert individually to prevent his/her judgment effected by other experts and the research purpose which does not have any conflict of interest will be informed. Then we explained the connotation of the 14 criteria (see Table 1 for details) and the significance of each blank in the questionnaire. After that, the experts will fill out the questionnaires and translated their subjective assessment about the importance of the criteria into numbers 1–5. We can have some auxiliary questions and answers during the process. Finally, the original results of seven experts were obtained, one of which were presented in Table 3.

**Table 3:** Sample assessment for criteria of one expert.

	C1	C2	C3	C4	C5	C6	C7	C8	C9	C10	C11	C12	C13	C14
C1	0	2	4	3	4	4	1	4	3	3	2	0	3	2
C2	**2**	0	4	3	2	4	3	4	2	3	2	3	3	3
C3	2	2	0	3	3	4	4	4	2	2	2	4	1	2
C4	3	4	4	0	4	2	4	3	2	3	0	1	4	3
C5	1	2	2	3	0	4	4	4	0	1	2	3	3	0
C6	1	2	3	3	3	0	3	2	4	1	2	4	1	2
C7	2	2	3	3	0	1	0	3	1	1	2	4	4	4
C8	2	2	1	2	0	1	4	0	4	2	1	4	2	4
C9	1	1	0	0	2	1	1	1	0	2	2	1	0	0
C10	3	2	2	1	0	2	2	1	1	0	0	1	0	1
C11	2	0	0	1	0	2	1	1	2	1	0	1	3	1
C12	3	3	2	1	2	0	1	1	0	4	4	0	1	4
C13	2	2	2	3	0	0	2	1	2	4	4	0	0	3
C14	2	3	0	0	2	0	0	0	4	4	4	4	3	0

Then, the original data are processed by utilizing formula (1)–(8) according to the CFCS method, and finally, the direct influence matrix for the influencing factors of big data on platform business model is determined, as shown in Table 4. The number in the direct influence matrix shows the degree of direct influence between corresponding elements.

**Table 4:** The direct influence matrix of big data on platform business model.

	**C1**	**C2**	**C3**	**C4**	**C5**	**C6**	**C7**	**C8**	**C9**	**C10**	**C11**	**C12**	**C13**	**C14**
**C1**	0.0000	0.2296	0.4609	0.2704	0.4609	0.4609	0.1071	0.4881	0.4065	0.2840	0.2704	0.0119	0.2160	0.2160
**C2**	0.2160	0.0000	0.4609	0.2704	0.2568	0.4609	0.2160	0.4609	0.2704	0.2840	0.2704	0.2296	0.2296	0.2296
**C3**	0.1752	0.2432	0.0000	0.2704	0.2704	0.4881	0.4337	0.4609	0.1480	0.1480	0.0391	0.4881	0.1888	0.2160
**C4**	0.2568	0.2840	0.4609	0.0000	0.4337	0.2704	0.4881	0.2840	0.2024	0.2840	0.0119	0.1888	0.4337	0.2840
**C5**	0.1752	0.2024	0.2568	0.2840	0.0000	0.4609	0.4609	0.4337	0.0119	0.0391	0.2704	0.2432	0.2296	0.0119
**C6**	0.2024	0.2024	0.2432	0.1888	0.1752	0.0000	0.2704	0.0527	0.4065	0.1071	0.1888	0.4609	0.2296	0.2024
**C7**	0.1616	0.1888	0.2024	0.2024	0.0119	0.1752	0.0000	0.2160	0.1616	0.1071	0.2024	0.4881	0.4337	0.4609
**C8**	0.1752	0.1480	0.1071	0.1480	0.0119	0.1207	0.4337	0.0000	0.4609	0.1888	0.1616	0.4337	0.2432	0.4881
**C9**	0.1207	0.0527	0.0119	0.0119	0.1888	0.1071	0.1071	0.0391	0.0000	0.1888	0.2704	0.1071	0.0119	0.0255
**C10**	0.2432	0.1616	0.1616	0.1071	0.0119	0.1616	0.1616	0.1071	0.1071	0.0000	0.0119	0.1207	0.0119	0.1071
**C11**	0.1616	0.0119	0.0119	0.1071	0.0119	0.1616	0.1071	0.1071	0.1888	0.1071	0.0000	0.1071	0.2160	0.1071
**C12**	0.2296	0.1888	0.2024	0.0935	0.1616	0.0119	0.1344	0.1344	0.0119	0.4881	0.4609	0.0000	0.1207	0.1752
**C13**	0.1480	0.1480	0.1480	0.2568	0.0119	0.0119	0.1752	0.1616	0.1752	0.4609	0.4881	0.0799	0.0000	0.2432
**C14**	0.1752	0.2296	0.0255	0.0119	0.2160	0.0119	0.0119	0.0119	0.4609	0.4881	0.4609	0.2568	0.2296	0.0000

Normalization is the normal operation of standardizing things. The direct influence matrix is standardized to obtain the standardized direct influence matrix by using formula (9). Then, according to the formula *T = G(E-G)*^*-1*^, MATLAB software is used for matrix calculation, and the comprehensive influence matrix was obtained, as shown in Table 5. The comprehensive influence matrix increases the indirect relation between criteria and can accurately reflect the comprehensive relation between criteria.

**Table 5:** The comprehensive influence matrix of big data on platform business model.

	**C1**	**C2**	**C3**	**C4**	**C5**	**C6**	**C7**	**C8**	**C9**	**C10**	**C11**	**C12**	**C13**	**C14**
**C1**	0.1552	0.1976	0.2721	0.2034	0.2440	0.2884	0.2244	0.2938	0.2876	0.2574	0.2532	0.2123	0.2225	0.2248
**C2**	0.2051	0.1445	0.2714	0.2013	0.1941	0.2819	0.2442	0.2837	0.2539	0.2622	0.2545	0.2623	0.2255	0.2307
**C3**	0.1895	0.1966	0.1611	0.1952	0.1904	0.2753	0.2849	0.2745	0.2150	0.2267	0.1988	0.3165	0.2125	0.2250
**C4**	0.2201	0.2189	0.2821	0.1488	0.2404	0.2473	0.3148	0.2562	0.2402	0.2729	0.2069	0.2609	0.2834	0.2525
**C5**	0.1721	0.1701	0.2035	0.1872	0.1117	0.2545	0.2746	0.2527	0.1640	0.1739	0.2268	0.2390	0.2084	0.1617
**C6**	0.1623	0.1534	0.1791	0.1451	0.1432	0.1273	0.1992	0.1423	0.2296	0.1773	0.1954	0.2553	0.1797	0.1740
**C7**	0.1561	0.1536	0.1688	0.1487	0.1036	0.1613	0.1359	0.1781	0.1823	0.1912	0.2079	0.2639	0.2315	0.2426
**C8**	0.1567	0.1406	0.1410	0.1304	0.1010	0.1450	0.2302	0.1223	0.2500	0.2042	0.1952	0.2494	0.1836	0.2461
**C9**	0.0766	0.0545	0.0522	0.0447	0.0843	0.0800	0.0816	0.0628	0.0569	0.1022	0.1239	0.0848	0.0542	0.0561
**C10**	0.1240	0.1010	0.1126	0.0846	0.0641	0.1155	0.1177	0.1033	0.1080	0.0860	0.0838	0.1160	0.0760	0.1015
**C11**	0.0933	0.0526	0.0597	0.0730	0.0499	0.0953	0.0887	0.0839	0.1139	0.0991	0.0740	0.0934	0.1116	0.0875
**C12**	0.1487	0.1293	0.1461	0.1049	0.1157	0.1095	0.1417	0.1395	0.1135	0.2340	0.2223	0.1170	0.1293	0.1440
**C13**	0.1293	0.1188	0.1304	0.1403	0.0804	0.1039	0.1498	0.1401	0.1549	0.2299	0.2286	0.1336	0.1033	0.1616
**C14**	0.1304	0.1304	0.0944	0.0775	0.1238	0.0995	0.1007	0.0990	0.2105	0.2304	0.2238	0.1611	0.1421	0.0890

According to formulas (10)–(13), influence degree, affected degree, centrality and causality are calculated as shown in Table 6. Influence degree is the sum of the rows in the matrix T that represents the comprehensive influence value of the corresponding row factor on all other factors. Affected degree is the sum of the columns in the matrix T that indicates the comprehensive affected value of the corresponding column factor on all other factors.

**Table 6:** Comprehensive influence matrix analysis.

Factor	Influence degree	Affected degree	Centrality	Causality
**C1**	3.3368	2.1194	5.4561	1.2174
**C2**	3.3155	1.9618	5.2773	1.3537
**C3**	3.1620	2.2745	5.4365	0.8875
**C4**	3.4453	1.8852	5.3306	1.5601
**C5**	2.8002	1.8467	4.6469	0.9536
**C6**	2.4632	2.3848	4.8480	0.0784
**C7**	2.5256	2.5885	5.1141	-0.0629
**C8**	2.4956	2.4322	4.9278	0.0634
**C9**	1.0147	2.5805	3.5952	-1.5658
**C10**	1.3941	2.7474	4.1415	-1.3533
**C11**	1.1759	2.6950	3.8709	-1.5191
**C12**	1.9954	2.7655	4.7609	-0.7701
**C13**	2.0049	2.3635	4.3684	-0.3586
**C14**	1.9127	2.3970	4.3097	-0.4843

**Fig 1:**
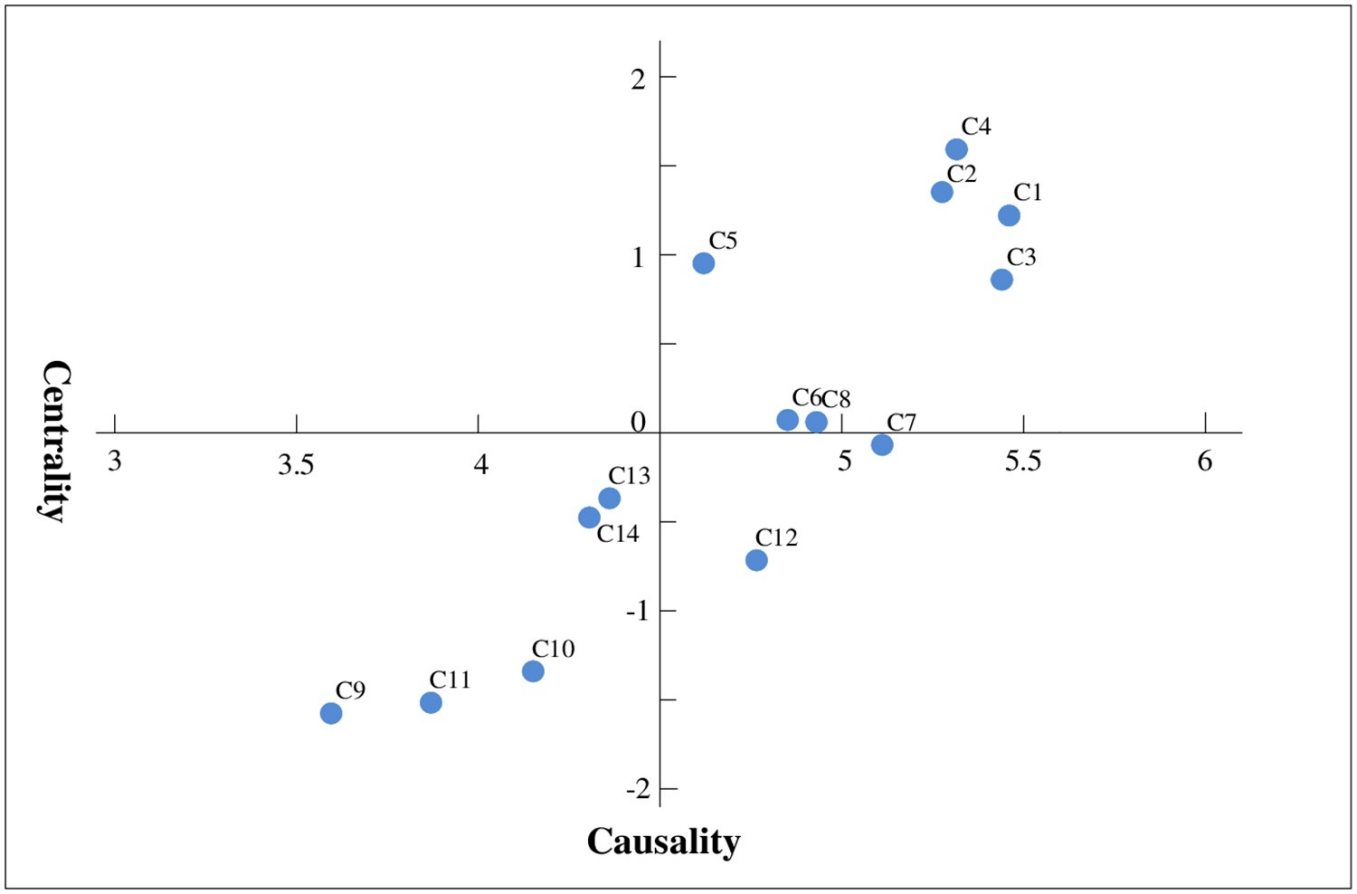
DEMATEL causal diagram.

Causality represents the influence degree of the criterion on the other criteria. Depending on whether causality is greater than or less than 0, 14 criteria are divided into a cause set and a result set. As seen from Table 5, there are 7 criteria in the cause set, including market segmentation(C1), value content(C2), information management(C3), community building(C4), technical support(C5), customer attraction(C6) and customer development(C8). Among them, market segmentation(C1), value content(C2) and community building(C4) are the main drivers. C1, C2 and C4 have corresponding influence degrees of 1.2174, 1.3537 and 1.5601, respectively, which are the three factors with the largest influence degrees, indicating that these three factors have the greatest influence on other factors. This is because the construction of a sustainable development system of platform enterprises under big data is inseparable from value proposition and community building, and value proposition will greatly affect its implementation and development. Community building is conducive to the establishment and improvement of a new model of information resource sharing mechanisms to facilitate the management of the platform. Therefore, the relevant measures should be taken into consideration when exploring the applications of big data on the platform business model. To make the causal relationship between the criteria clearer, a causal relationship diagram is made, as shown in Fig 1.

The others are 7 result elements, including relationship management(C7), pricing(C9), media advertising(C10), financial products(C11), privacy security(C12), symbiosis(C13) and legality(C14). These result elements have a weaker impact on the platform business model based on big data but are more likely to be affected by other factors to make changes. Therefore, proper attention and control should be paid to actual management to help improve the management effect.

Centrality indicates the position of the criteria in the system and the role they play in the s system of e-commerce platform business model based on big data. According to the degree of centrality, the criteria are C1, C3, C4, C2, C7, C8, C6, C12, C5, C13, C14, C10, C11 and C9 in descending order. More attention should be paid to criteria with higher centrality, such as market segmentation(C1), information management(C3) and community building (C4).

ISM is used to construct the system into a multi-level hierarchical structure model. The overall influence matrix H obtained by formula (14) is shown in Table 7, already considering the influence of criteria on itself.

**Table 7:** Overall influence matrix.

	**C1**	**C2**	**C3**	**C4**	**C5**	**C6**	**C7**	**C8**	**C9**	**C10**	**C11**	**C12**	**C13**	**C14**
**C1**	1.1552	0.1976	0.2721	0.2034	0.2440	0.2884	0.2244	0.2938	0.2876	0.2574	0.2532	0.2123	0.2225	0.2248
**C2**	0.2051	1.1445	0.2714	0.2013	0.1941	0.2819	0.2442	0.2837	0.2539	0.2622	0.2545	0.2623	0.2255	0.2307
**C3**	0.1895	0.1966	1.1611	0.1952	0.1904	0.2753	0.2849	0.2745	0.2150	0.2267	0.1988	0.3165	0.2125	0.2250
**C4**	0.2201	0.2189	0.2821	1.1488	0.2404	0.2473	0.3148	0.2562	0.2402	0.2729	0.2069	0.2609	0.2834	0.2525
**C5**	0.1721	0.1701	0.2035	0.1872	1.1117	0.2545	0.2746	0.2527	0.1640	0.1739	0.2268	0.2390	0.2084	0.1617
**C6**	0.1623	0.1534	0.1791	0.1451	0.1432	1.1273	0.1992	0.1423	0.2296	0.1773	0.1954	0.2553	0.1797	0.1740
**C7**	0.1561	0.1536	0.1688	0.1487	0.1036	0.1613	1.1359	0.1781	0.1823	0.1912	0.2079	0.2639	0.2315	0.2426
**C8**	0.1567	0.1406	0.1410	0.1304	0.1010	0.1450	0.2302	1.1223	0.2500	0.2042	0.1952	0.2494	0.1836	0.2461
**C9**	0.0766	0.0545	0.0522	0.0447	0.0843	0.0800	0.0816	0.0628	1.0569	0.1022	0.1239	0.0848	0.0542	0.0561
**C10**	0.1240	0.1010	0.1126	0.0846	0.0641	0.1155	0.1177	0.1033	0.1080	1.0860	0.0838	0.1160	0.0760	0.1015
**C11**	0.0933	0.0526	0.0597	0.0730	0.0499	0.0953	0.0887	0.0839	0.1139	0.0991	1.0740	0.0934	0.1116	0.0875
**C12**	0.1487	0.1293	0.1461	0.1049	0.1157	0.1095	0.1417	0.1395	0.1135	0.2340	0.2223	1.1170	0.1293	0.1440
**C13**	0.1293	0.1188	0.1304	0.1403	0.0804	0.1039	0.1498	0.1401	0.1549	0.2299	0.2286	0.1336	1.1033	0.1616
**C14**	0.1304	0.1304	0.0944	0.0775	0.1238	0.0995	0.1007	0.0990	0.2105	0.2304	0.2238	0.1611	0.1421	1.0890

Then, the threshold λ by formula (15) can be calculated and the research will get λ = α+β = 0.1678+0.0677 = 0.2355. The value higher than λ in the overall influence matrix means row factors affect column factors and the corresponding position is marked as 1, while the value lower than λ means row factors do not affect column factors and the corresponding position is marked as 0. Finally, the reachability matrix can be obtained by formula (16) and (17). The reachability matrix in the Table 8 determines whether the two factors impact each other.

**Table 8:** Reachability matrix.

	**C1**	**C2**	**C3**	**C4**	**C5**	**C6**	**C7**	**C8**	**C9**	**C10**	**C11**	**C12**	**C13**	**C14**
**C1**	1	0	1	1	1	1	1	1	1	1	1	1	1	1
**C2**	1	1	1	0	0	1	1	1	1	1	1	1	1	1
**C3**	0	0	1	0	0	1	1	1	1	1	0	1	1	1
**C4**	1	1	1	1	1	1	1	1	1	1	1	1	1	1
**C5**	0	0	1	0	1	1	1	1	0	0	1	1	1	0
**C6**	0	0	0	0	0	1	0	0	1	0	0	1	0	0
**C7**	0	0	0	0	0	0	1	0	0	0	1	1	1	1
**C8**	0	0	0	0	0	0	1	1	1	1	0	1	0	1
**C9**	0	0	0	0	0	0	0	0	1	0	0	0	0	0
**C10**	0	0	0	0	0	0	0	0	0	1	0	0	0	0
**C11**	0	0	0	0	0	0	0	0	0	0	1	0	0	0
**C12**	0	0	0	0	0	0	0	0	0	1	1	1	0	0
**C13**	0	0	0	0	0	0	0	0	0	1	1	0	1	0
**C14**	0	0	0	0	0	0	0	0	1	1	1	0	0	1

The first-level decomposition structure is obtained from the reachability matrix and formula (18), as shown in Table 9.

**Table 9:** First-level decomposition structure.

*i*	*L(f*_*i*_*)*	*P(f*_*i*_*)*	*C(f*_*i*_*) = L(f*_*i*_*)∩P(f*_*i*_*)*
C1 market segmentation	1,3,4,5,6,7,8,9,10,11,12,13,14	1,2,4	1,4
C2 value content	1,2,3,6,7,8,9,10,11,12,13,14	2,4	2
C3 information management	3,6,7,8,9,10,12,13,14	1,2,3,4,5	3
C4 community building	1,2,3,4,5,6,7,8,9,10,11,12,13,14	1,4	1,4
C5 technical support	3,5,6,7,8,11,12,13	1,4,5	5
C6 customer attraction	6,9,12	1,2,3,4,5,6	6
C7 relationship management	7,11,12,13,14	1,2,3,4,5,7,8	7
C8 customer development	7,8,9,10,12,14	1,2,3,4,5,8	8
C9 pricing	9	1,2,3,4,6,8,9,14	9
C10 media advertising	10	1,2,3,4,8,10,12,13,14	10
C11 financial products	11	1,2,4,5,7,11,12,13,14	11
C12 Privacy security	10,11,12	1,2,3,4,5,6,7,8,12	12
C13 symbiosis	10,11,13	1,2,3,4,5,7,13	13
C14 legality	9,10,11,14	1,2,3,4,7,8,14	14

**Fig 2:**
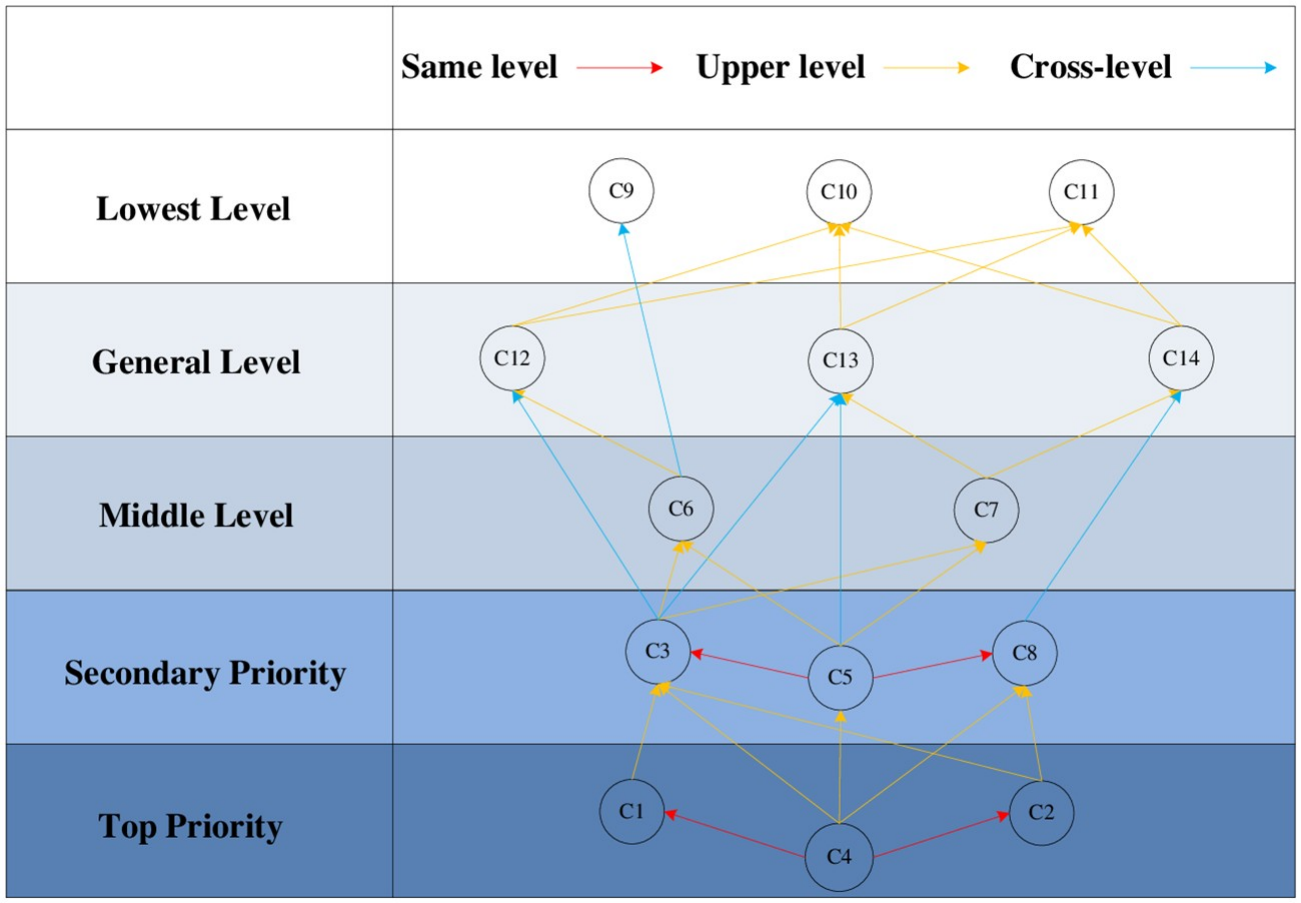
ISM model structure.

As seen from Table 9, the reachable set and the common set intersect in C9, C10 and C11, so C9, C10 and C11 constitute the first-level influencing factors. The rows and columns mapped by influence factors C9, C10, and C11 in matrix M are deleted to obtain a higher-level decomposition matrix, and the above process is repeatedly performed. After multiple hierarchical divisions, the factor set Nq (q = 1, 2, …, 9) of each layer is finally obtained: first-level node N1 = {C9, C10, C11}; second-level node N2 = {C12, C13, C14}; third-level node N3 = {C6, C7}; fourth-level node N4 = {C8}; fifth-level node N5 = {C3}; sixth-level node N6 = {C5}; seventh-level node N7 = {C1}; eighth-level node N8 = {C2}; and ninth-level node N9 = {C4}. Based on the above analysis, an ISM model is presented in Fig 2.

From the ISM model structure of the influence factor, we can see that market segmentation(C1), value content(C2) and community building(C4) are the root causes of the impact of big data on the platform business model. Thus, the way in which to effectively control the tracking and control of big data becomes a key focus. Platform enterprises should identify target customers according to customers’ browsing and purchasing history and decide whether to develop their own products or own logistics systems according to the evaluation of goods purchased by customers. This also helpful for enterprises to provide more targeted services. While value acquisition (including pricing(C9), media advertising(C10) and financial products(C11)) lays at the last level, it reflects the ultimate value flow of platform business model in big data era.

In conclusion, the factors that affect the application of big data on the platform business model are very complex, and five aspects interact with each other. However, different factors have different influence modes, mechanisms and degrees of action, thus forming a systematic integration framework of platform business model under big data.

## 5. Discussion

Because the existing framework of platform business model under big data is indistinct, this paper attempts to explore the path of platform business model based on big data. This study systematically proposed a set of criteria about the development of platform business model and constructed a hierarchical model to compensate for the shortage of such research in the extant literature.

Market segmentation and value content are at the first level in the model framework; therefore, value proposition is still the core of the platform business model under big data. The clear value proposition, an effective method for enterprises to transfer their core identity and values [64], is not only a cornerstone of strategy [65] but also a useful tool for product marketing [66]. The value proposition of platform enterprises under big data is not different from that of traditional enterprises, which are not affected by big data and are still the core links of enterprises. The difference is that, after big data is embedded, the value proposition of platform enterprises is richer. Platform enterprises under big data can constantly adjust their value propositions based on the dynamic needs of customers and seek the blue ocean market to attract more customer groups, thus improving their comprehensive competitiveness. At the first level, this conclusion not only unifies the platform business model and traditional business model but also verifies that value propositions under big data remain the core of the business model, which promotes the consistency of the overall theory of the business model.

At the same time, community building is also at the first level, which has become an important new aspect of the development of the business models of platform enterprises under big data. In traditional enterprises, the importance of the ecosystem is emphasized [67], but insufficient attention has been paid to it, which has something to do with the preference for product production. However, in the context of big data, community building has become a key activity in the value production of platform enterprises [30]. Community building is conducive to the reasonable integration of various resources [48] and the establishment and improvement of a new model of information resource sharing mechanisms [68] to facilitate the management of the platform by enterprises. This conclusion reflects the characteristics of platform enterprises under big data and renders the advantages brought about by big data more obvious.

In the entire platform business model system, the first level plays a fundamental role, while the information technology and information management of the second level are the continuity points for platform enterprises to attract customers, which becomes more important than in traditional enterprises. In traditional enterprises, information management and information technology, as support functions, serve the main functions and become help improve the efficiency of the main functions. However, for platform enterprises, especially after big data technology is embedded, the value production process of the entire business model is more dependent on the improvement of the information system and the application of information technology. The improvement of the information technology system provides a more ideal platform browsing experience for customers [48], which is conducive to better maintaining customer relations [52], thus attracting more customers to the platform [49], consistent with the results in the existing literature. The technical advantages brought about by big data are reflected in the front end of value production in platform enterprises, while in traditional enterprises, there is a time lag [69], so it can see that the two have certain differences.

Value marketing and value network are closely linked. A prerequisite of value network construction is value marketing. Only by gaining the trust of customers and conducting effective customer relationship management can we build a better and more stable value network [52]. Marketing based on big data is more targeted and can better grasp user behaviors and psychological preferences to adopt a more effective relationship management approach [52, 70]. In addition, against the background of big data, customer development in value marketing becomes more important, which is conducive to the sustainable development and long life of enterprises. At the same time, new criteria of the value network, including the three features of symbiosis, legality and privacy security, are developed. The development of criteria is guided by the construction of ecosystems, emphasizing that the optimization of ecological networks and mutual recognition among stakeholders are the keys to network optimization. Value network construction is also the last link before value acquisition in the platform business model under big data. Value marketing and value networks become the only ways to obtain value.

Value acquisition is the last link of the platform business model and is also at the last level. Compared with traditional enterprises that set prices after developing products and then use marketing methods to promote them [71, 72], the business model of platform enterprises under big data is different. Reasonable pricing and traffic attraction become the final links, based on the perfect value network and customer flow. Whether to develop self-supporting products and how to effectively undertake advertiser injection for platform enterprises also depend on whether the value network is mature [73]. At the same time, value acquisition under precision marketing also enables platform enterprises to select different pricing schemes according to customer characteristics. All of these aspects are very different from those of traditional business models.

## 6. Conclusions

Currently, most studies pay more attention to the impact of big data on a certain link in the value chain of platform enterprises and tend to ignore the mutual coordination and limitations of various links in the value chain, which is not conducive to the overall operation of platform enterprises and the overall design of the platform business model. In addition, in the application of big data to platform enterprises, most studies adopt the method of case analysis, focusing on the role of big data in a single well-known platform enterprise, thus lacking a universal integration framework. Compared with previous studies, this paper systematically explores the overall impact of big data on the platform business model and considers the synergy and limitations between modules of the platform business model, revealing the hierarchical path of the platform business model under big data, which is of great significance in compensating for the shortage of existing research on this topic. This research comprehensively applies fuzzy, DEMATEL and ISM as integrated research methods, not only eliminating the influence of experts’ subjective factors but also considering the interactions between different factors and clarifying the different levels and paths, thus providing important information for the realization of the above research work in this paper. In addition, the outbreak of COVID-19 brings new challenges to E-commerce platforms, putting E-commerce industry in a crisis. At the same time, it also provides E-commerce enterprises an opportunity to innovate business models, making "new online models" possible. This study not only sorts out and constructs the platform business model innovation system in the big data environment, but also has reference significance for the business model innovation of small and medium-sized e-commerce enterprises facing current challenges by constructing the hierarchical theoretical framework.

There are still certain limitations to this study. First, although the proposed criteria have been selected through the extensive literature review, it is still insufficient to cover all possible attributes; thus, further exploration and improvement in future research are needed. Second, expert committee was consisted with the experts worked in the platform enterprises, experts in other fields related to electronic technology, especially big data, should be included in the committee for increasing the scope and the applicable boundaries. Third, the relationships and degrees of influence among criteria are processed and analyzed based on the data information of questionnaires completed by experts. Although fuzzy set theory is used in this paper to solve the problem of experts’ subjective bias, there are still errors that cannot be completely eliminated, which could have a certain impact on the research results of this paper. In addition, this study could also use other statistical tools, such as structural equation models, to explore more influencing factors and perform the statistical verification of the model.

## Supporting information

S1 Data (XLSX)
